# Newly manufactured Marukome MK-34-1 miso with angiotensin-converting enzyme inhibitory activity and its antihypertensive effects in genetic hypertensive rat models

**DOI:** 10.1038/s41440-018-0197-z

**Published:** 2019-01-10

**Authors:** Hiroe Sakuyama Tomari, Misa Uchikawa, Aki Yamazaki, Satomi Hirabayashi, Shoko Yamakawa, Manabu Kitagawa, Minami Yamada, Seiki Itou, Tetsuro Yamamoto, Yoshio Uehara

**Affiliations:** 1 Division of Clinical Nutrition, Faculty of Home Economics, Kyoritsu Women’s University, Tokyo, Japan; 2 Marukome Co. Ltd., Nagano, Japan; 3 TTC Co. Ltd., Tokyo, Japan

**Keywords:** Miso, ACE, SHRSP, Dahl rats, Hypertension

## Abstract

We newly manufactured miso rich in angiotensin-converting enzyme (ACE) inhibitory activity (Marukome MK-34-1, shinki miso) and investigated its antihypertensive properties in rat models of genetic hypertension. ACE inhibitory activity was tenfold higher in shinki miso than in commercially available Marukome Nenrin miso (nenrin miso). The inhibitory activity of shinki miso was confined to <3 kDa fractions and was detected in several fractions with high polarity by C_18_ high-performance liquid chromatography. Systolic blood pressure (SBP) increased age-dependently in stroke-prone spontaneously hypertensive rats (SHRSP/Izm) given a 0.6% (w/v) NaCl solution (salt solution group) that matched the salt content of the miso solutions. This SBP increase was attenuated in both the 5% nenrin and 5% shinki miso solution groups compared to the salt solution group. The reduction in SBP was greater in rats fed shinki than in rats fed nenrin miso. Similarly, in a salt-induced hypertension model with Dahl rats, the 5% nenrin miso solution attenuated the rising SBP observed in the salt solution group. Moreover, combining 5% nenrin miso with 5% shinki miso (2:1, v/v) (awase miso group) significantly decreased the SBP per gram salt intake by 8% compared with the nenrin miso treatment. However, there were no differences in urinary Na excretion between the nenrin and awase miso groups. In conclusion, we produced a new miso with potent ACE inhibitory activity that reduced spontaneous and salt-induced hypertension. These results suggest that salt sensitivity is decreased by the addition of shinki miso to nenrin miso.

## Introduction

Miso is a traditional Japanese food whose consumption dates to 700 AD. It is usually produced from soybeans with malted rice and salt. One serving of miso soup contains 1–2 g of salt. Therefore, traditional miso soup has long been assumed to be the reason for the high incidence of salt-sensitive hypertension, which can potentially cause cerebral stroke, in Japan.

We recently demonstrated that miso soup attenuates salt-induced hypertension in Dahl rats prone to salt-induced hypertension (Dahl S rats) [1, 2]. This was attributed to natriuresis or vasodilation induced by miso soup ingredients. Intraperitoneal or subcutaneous administration of a very small amount of miso extract decreased the blood pressure (BP) in Dahl S rats [3], an effect that has also been investigated in clinical settings. However, in a human intervention trial, two daily servings of miso soup (3.8 g salt/day) for 3 months did not affect the systolic blood pressure (SBP) or diastolic blood pressure (DBP) of subjects with normotension or stage 1 hypertension [4]. Moreover, a 5-year cross-sectional observation found no association between the frequency of miso soup intake and the SBP and DBP of subjects with normal or high normal BP [5].

Some studies have suggested that miso contains compounds that attenuate the BP response to high salt intake [1–5]. Although these hypotensive factors remain to be identified, it can be supposed that increasing hypotensive factor content would promote miso consumption as a health food. In fact, enzymatically digested soybeans were found to reduce BP, and nicotianamine extracted from soybeans inhibited angiotensin-converting enzyme (ACE) activity [6, 7]. Our preliminary experiments showed that miso crude extract inhibits ACE activity in vitro by 10–20% (unpublished data).

We speculated that miso with potent ACE inhibitory activity attenuates hypertension to a greater extent than regular commercially available miso, particularly hypertension associated with enhancement of the renin–angiotensin system (RAS). To test this hypothesis, we produced a novel type of miso (Marukome MK-34-1) with potent ACE inhibitory activity and examined its hypotensive effects in rat models of spontaneous and salt-induced hypertension.

Long-term diuresis is associated with Na imbalance and decreased body fluid space. Such disruption leads to enhanced RAS activity with attenuation of BP reduction. Here, to examine whether shinki miso works as an ACE inhibitor, we tested the possible synergistic effects of regular miso (Marukome Nenrin) and miso with potent RAS inhibitory activity.

## Methods

### Characterization of hypotensive properties of shinki miso in a spontaneous hypertension model, SHRSP/Izm

Male stroke-prone spontaneously hypertensive/Izumo rats (SHRSP/Izm) (8 weeks old, *n* = 30) were purchased from Sankyo Laboratory (Tokyo). The rats were randomly divided into three equal-sized groups (ten rats in each group) that were given (1) 5% (w/v) Marukome MK-34-1 miso (shinki miso, Nagano) solution with potent ACE inhibitory activity (0.6% NaCl, w/v) (shinki group); (2) 5% (w/v) commercially available Marukome Nenrin miso solution (0.6% NaCl, w/v) (nenrin group); or (3) 0.6% NaCl (w/v) solution (salt solution group). The rats were all fed regular chow (0.6% NaCl, w/w) (Oriental Kobo MF, Tokyo) for 4 weeks. Miso or salt solutions and chow were freely available throughout the experiment.

### Combined effects of shinki miso and nenrin miso on salt-induced hypertension in Dahl rats

Male Dahl salt-sensitive (Dahl S/Iwai) rats (4 weeks old, *n* = 24) were purchased from Sankyo Laboratory and were fed regular chow (0.6% NaCl, w/w) chow and given 1% NaCl (w/v) solution as drinking water. After 3 weeks, the rats were randomly divided into three equal-sized groups (eight rats in each group) that were given (1) 0.6% NaCl (w/v) solution (salt solution group); (2) a 2:1 (v/v) combination of 5% nenrin miso (0.6% NaCl, w/v) and 5% shinki miso with potent ACE inhibitory activity (0.6% NaCl, w/v) solutions (awase group); or (3) 5% nenrin miso solution (0.6% NaCl, w/v) (nenrin group). The rats were fed regular chow (0.6% NaCl, w/w) (Oriental Kobo MF) for 4 weeks. Miso or salt solutions and chow were freely available throughout the experiment. Food consumption was measured every 2–3 days.

### Preparation of miso solution

We utilized 5% (w/v) nenrin miso solutions because we had reported that conventional miso solutions with concentrations >5% (w/v) attenuate salt-induced hypertension in Dahl S rats [1, 2]. The salt concentration in the 5% (w/v) nenrin solution was determined using a Na Meter Model TS-999i (Toko Chemical Laboratories Co. Ltd., Tokyo). Then, shinki miso was manufactured to make the salt concentration equal to that in nenrin miso.

Miso dissolved in distilled water (5%, w/v) was homogenized using an ultrasonicator. Then, the homogenate was autoclaved at 120 °C for 1 h. Insoluble residues were removed using a cotton filter, and the soluble fraction was used as the miso solution. The solution was stored at 4 °C until use. The preparation was repeated every 2–3 days.

### BP measurement

SBP was measured weekly by the tail-cuff method as previously described but with a modified detection system (Model KN-210-1; Natsume Seisakujo, Tokyo) [8]. The same investigator measured the SBP of all animals in a quiet, warm room between 10:00 and 14:00 h.

### Sample collection

At the end of the experiment, the rats were placed in a metabolic cage to collect 24-h urine samples [1, 2]. The volume of salt and miso solutions consumed by the rats was measured every 1–3 days. Each animal was anesthetized with pentobarbital sodium (i.p., 75 mg/kg body weight), and then the kidney, heart, and aortic wall along with the blood were collected. The aortic wall samples (1 cm segments) were obtained from the descending thoracic aorta just distal to the aortic arch and then weighed. Urine and blood samples were stored at −80 °C until assayed.

### Characterization of shinki miso

A batch of regular commercially available nenrin miso was supplied by Marukome Co. Ltd., and stored at −20 °C until use to prevent further fermentation. The new shinki miso with potent ACE inhibitory activity was originally produced by Marukome [9]. The main characteristics are listed in Table 1. The new shinki miso was prepared in the same manner as nenrin miso using soybeans, malted rice, and salt but with a much shorter fermentation period (5 vs 46 days). Additionally, different species of Koji starter were used for the two types of miso, the details of which cannot be disclosed due to the patent policy.

**Table 1:** Characterization of Marukome shinki miso

	Shinki	Nenrin
Materials	Soybeans, rice, salt, ethanol, free of yeast	Soybeans, rice, salt, ethanol, yeast
Percentage of malted rice^a^	50%	70%
Species of Koji starter	Not open	Aspergillus oryzae
Salt content	12.2%	12.1%
Fermentation term	5 days	46 days
ACE inhibitory activity^b^ (IC_50_)	0.23 mg/mL	2.5 mg/mL

Shinki, Marukome shinki miso rich in ACE inhibitory activity; nenrin, Marukome Nenrin miso

^a^Percentage of Koji starter was calculated according to the equation 100 × malted rice (g)/soybean (g)

^b^ACE inhibitory activity was determined according to the method previously reported [9–11]

### Evaluation of ACE inhibition

ACE activity was determined according to an established method [9–11]. Briefly, miso extract was mixed with standard ACE and 25 mM hippuryl-histidyl-leucine substrate in HEPES buffer (pH 8.3). The cleaved histidyl-leucine emitted a fluorescence signal (excitation/emission, 340/485 nm) in the presence of 0.2% *o*-phthalaldehyde in methanol. The inhibitory activity was calculated with the following equation: activity = 100 × (1−ACE activity with miso extract/ACE activity without miso). The half-maximal inhibitory concentrations (IC_50_s) were 0.23 and 2.5 mg/mL for new and regular miso, respectively.

### Measurement of urinary and blood parameters

Electrolytes in blood and urine were measured using an autoanalyzer (Model 736; Hitachi Co., Tokyo). Urinary protein concentrations were measured using a protein assay kit (Bio-Rad, Hercules), while urinary catecholamine was measured by high-performance liquid chromatography (HPLC; SRL Laboratories, Tokyo). Plasma and urine osmolality was measured by the standard molecular freezing point depression method (SRL Laboratories). Measurements were performed by investigators who were blinded to the group assignments.

### Evaluation of oxidative stress in the kidney

Since the RAS is closely associated with upregulation of oxidative stress, we investigated the relationship between oxidative stress and renal function in rats given miso solution. To assess oxidative stress in the kidney, we estimated lipid peroxide levels in kidney homogenates based on malondialdehyde (MDA) production [12, 13]. Briefly, kidney tissue was homogenized in ice-cold Dulbecco’s balanced phosphate-buffered saline using an ultrasonicator and centrifuged. A portion of the supernatant was incubated at 37 °C for 30 min. The generated lipid peroxides were converted to MDA with trichloroacetate. The product was coupled with thiobarbiturate, and the density was determined at a wavelength of 532 nm. Protein levels in the supernatant were assayed using a protein assay kit (Bio-Rad).

### Analysis of miso extract by C_18_ reversed-phase HPLC

Miso extract was analyzed by reversed-phase HPLC (COSMOSIL 5C18-AR-II; Naka-Arai Techs, Tokyo) with a C_18_ column (inner diameter, 6.0 × 150 mm). Briefly, the sample was loaded onto the column and eluted at 0.8 mL/min and 40 °C with a 0.1% trifluoroacetate solution and a 0–80% acetonitrile gradient for 50 min. The fractionated effluent was evaporated, and the residue was dissolved in phosphate-buffered solution to determine ACE inhibitory activity.

Moreover, we characterized the ACE inhibitory activity in fractions of shinki miso extracts with molecular weights <3, 3–0, 10–30, and >30 kDa produced with membrane sieves (adjusted to 100 mg extract/mL) (Merck Millipore Corporation, Darmstadt).

### Statistical analysis

All statistical analyses were performed using STATISTICA software (StatSoft, Tulsa). The values are expressed as the mean ± SD. Differences were assessed by one-way analysis of variance (ANOVA) followed by Fisher’s least significant difference post hoc test (LSD test), repeated measures ANOVA, or nonparametric Mann–Whitney *U* tests. *P* values < 0.05 were considered statistically significant.

### Guidelines for handling rats in experiments

The study protocol conformed to the institutional guidelines for experimental animal handling and was approved by the Animal Care Committee of the Kyoritsu Women’s University (#16002 and #17002). The experiments were conducted in accordance with the guidelines of the National Institutes of Health (Bethesda, MD, USA).

## Results

### ACE inhibitory activity in the new shinki miso

**Fig. 1:**
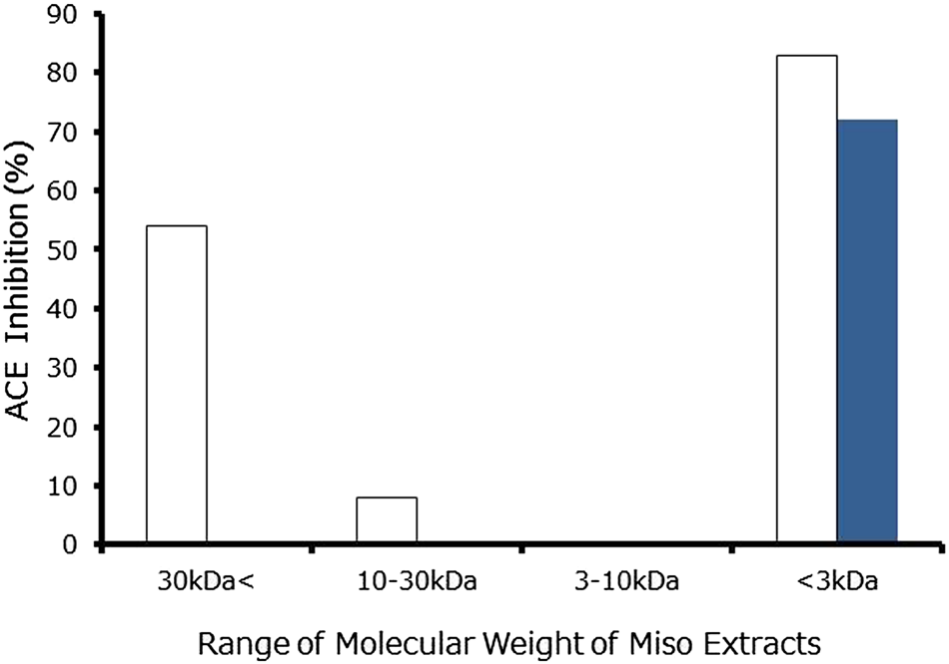
ACE inhibitory activity of shinki miso. Miso extracts were separated according to molecular weights using membrane sieves as described in the text. In this assay system, the concentrations of both miso extracts to be analyzed were well above the IC_50_ (100 mg/mL). The left open column represents the nenrin miso, and the right solid column represents the shinki miso rich in ACE inhibitory activity

**Fig. 2:**
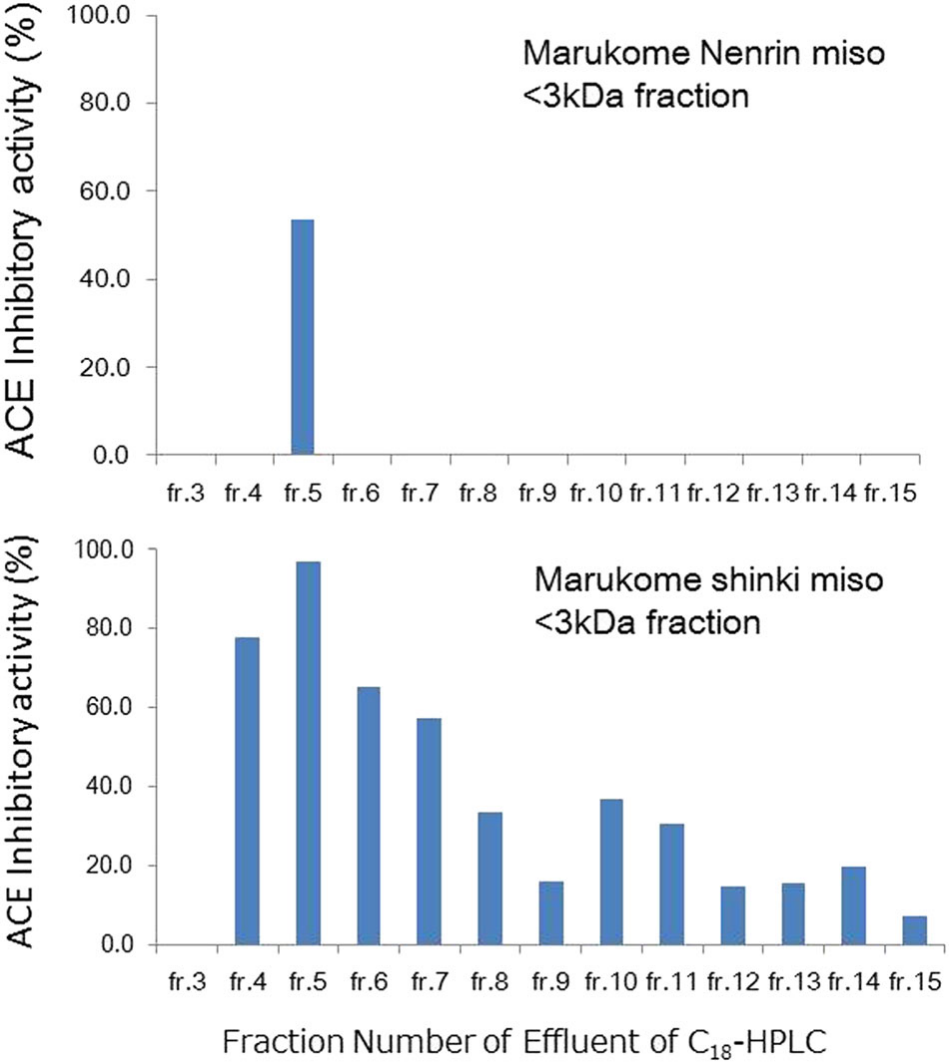
ACE inhibitory activity in fractionated effluents as determined by C_18_ reversed-phase HPLC. The upper graph represents <3 kDa nenrin miso extracts, and the lower graph represents <3 kDa extracts of shinki miso. The miso extracts (10 µL) were analyzed using C_18_ reversed-phase HPLC as described in the text

The IC_50_ of ACE inhibition by shinki miso crude extracts was one-tenth that of nenrin miso crude extracts (Table 1). We determined the ACE inhibitory activity in fractions of shinki miso extracts with molecular weights of <3, 3–10, 10–30, and >30 kDa (Fig. 1). For shinki miso, inhibitory activity was confined to the <3 kDa fraction, whereas for nenrin miso, inhibitory activity was observed in three fractions, although it was mostly in the <3 kDa fraction. We therefore further analyzed this fraction by C_18_ reversed-phase HPLC and found that the inhibitory activity was present in higher-polarity fractions of shinki miso extract but was limited to fraction 5 of nenrin miso (Fig. 2). In the shinki miso, we found several dipeptides with ACE inhibitory activity in HPLC-MS/NMR (mass-spectrophotometry/nuclear magnetic resonance) analysis (unpublished data). We have not determined a single substance responsible for the ACE inhibitory activity of shinki miso. Several substances may contribute to the apparent ACE inhibitory activity of shinki miso.

### Characterization of hypotensive properties of shinki miso in a spontaneous hypertension model, SHRSP/Izm

#### Miso consumption

**Fig. 3:**
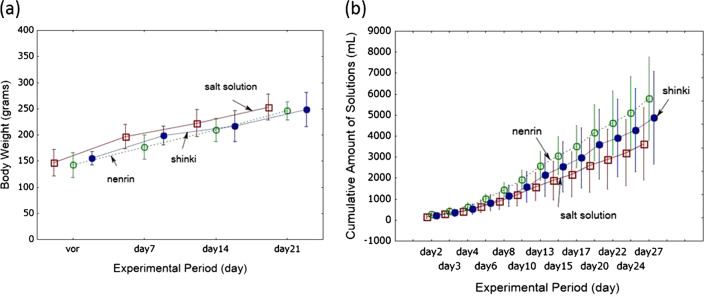
Body weight changes and cumulative amounts of solution consumption in the experimental groups. The body weight changes are shown in graph (**a**). The cumulative amounts of consumed miso and salt solutions are shown in graph (**b**). Open squares, salt solution group; open circles, nenrin miso group; closed circles, shinki miso group. The values in the graphs represent the means ± SD (*n* = 10 in each group). Group differences in graph (**a**) were analyzed using one-way analysis of variance (ANOVA) followed by Fisher’s least significant difference post hoc test (Fisher LSD test), and those in group (**b**) were analyzed using repeated measures analysis of variance. There were no group differences in body weights at day 14 and day 21. The cumulative amounts of miso or salt solution consumed during the experiment differed among the groups (*P* < 0.001). The difference between the salt solution and nenrin miso groups was significant (*P* < 0.025); however, the consumption of the shinki miso group did not differ from that of the nenrin miso group

The body weight of rats increased in an age-dependent manner in all experimental groups (Fig. 3a). There were no differences in body weight among the three groups during the experimental period. The cumulative amounts of miso or salt solution consumed are shown in Fig. 3b. Consumption was higher in the nenrin miso group than in the salt solution group but was lower in rats fed shinki than in rats fed nenrin miso (*P* < 0.001). As each solution contained 0.6% NaCl (w/v), the cumulative amount of salt obtained from drinking the solution was estimated to be 9.63 ± 2.19 g for the salt solution group, 15.31 ± 2.41 g for the nenrin miso group (*P* < 0.05 vs salt solution group), and 12.84 ± 2.75 g for the shinki miso group (*P* = 0.057 vs salt solution group).

#### Effects of shinki miso on systolic blood pressure in SHRSP/Izm

The organ weights are presented in Table 2. There were no differences in heart and kidney weights among the three experimental groups. In the nenrin miso group, the aortic wall weight was unchanged when compared to that in the salt solution group. In contrast, in the shinki miso group, the aortic wall weight was 8.7% lower than that in the salt solution group (*P* < 0.05).

**Table 2:** Organ weights in experimental groups

Group	Heart	Kidney	Aorta
(Units)	(g/100 g BW)	(g)	(mg/mm^2^)
Salt solution	0.440 ± 0.017	1.262 ± 0.085	0.149 ± 0.018
Nenrin	0.443 ± 0.026	1.240 ± 0.058	0.149 ± 0.014
Shinki	0.449 ± 0.016	1.272 ± 0.082	0.136 ± 0.006*,**

Salt solution, group given salt solution; nenrin, Marukome Nenrin miso group; and shinki, Marukome shinki miso with potent ACE inhibitory activity miso group. Values are expressed as means ± SD (*n* = 10 in each group). Differences were assessed by one-way analysis of variance (ANOVA) followed by Fisher’s least significant difference post-hoc test (LSD test)

*P  < 0.05 vs salt solution, **P  < 0.05 vs nenrin

**Fig. 4:**
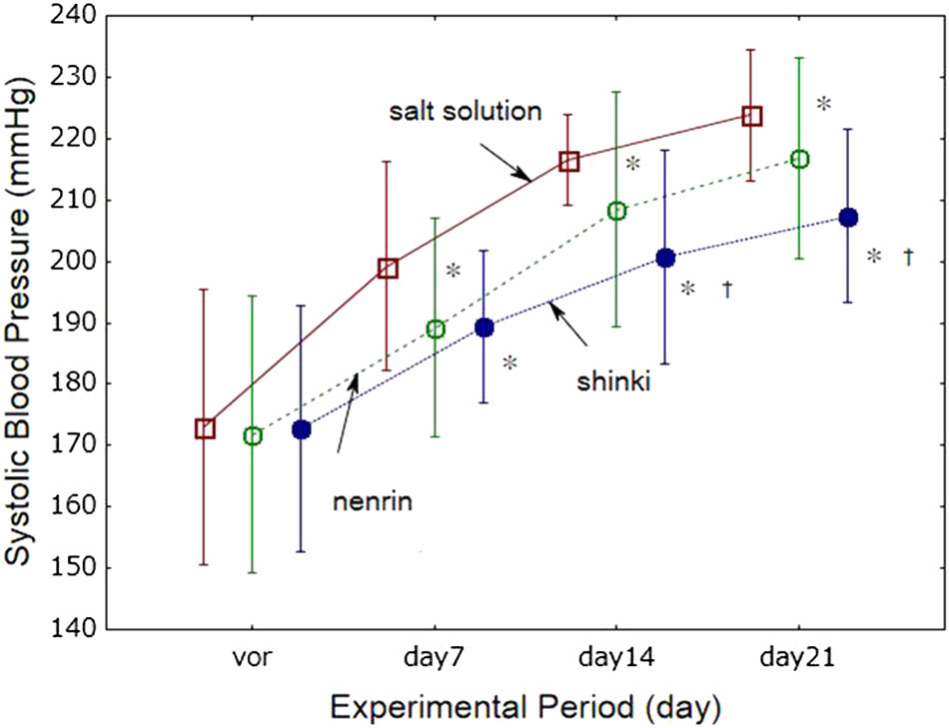
Effects of shinki miso on systolic blood pressure in SHRSP/Izm. Open squares, salt solution group; open circles, nenrin miso group; closed circles, shinki miso rich in ACE inhibitory activity group. The values in the graph represent the means ± SD (*n* = 10 in each group). Group differences were analyzed using repeated measures analysis of variance with Fisher’s LSD test (*P* < 0.001). *P < 0.05 vs salt solution group, †*P* < 0.05 vs nenrin miso group

SBP increased age-dependently in the three experimental groups (Fig. 4). Even though rats fed miso had a higher salt intake than those fed the salt solution, SBP was lower in the miso-fed animals. Moreover, SBP was 3.8% lower at week 2 and 4.1% lower at week 3 in rats that consumed shinki than in rats that consumed the nenrin miso solution. The SBP/cumulative salt intake was 13.37 ± 2.94 mmHg/g salt intake for the salt solution group, 7.98 ± 1.29 mmHg/g salt intake for the nenrin miso group (*P* < 0.0001 vs salt solution group), and 9.14 ± 2.09 mmHg/g salt intake for the shinki miso group (*P* < 0.001 vs salt solution group). There were no differences in salt sensitivity between the shinki miso and nenrin miso groups.

#### Effects of shinki miso on renal function in SHRSP/Izm

Blood electrolyte concentrations did not differ among the experimental groups (Table 3). Shinki miso influenced neither urinary nor fractional sodium excretion, whereas urinary sodium excretion was higher in the nenrin miso group than in the salt solution group. Shinki miso did not influence potassium clearance or urinary aldosterone excretion.

**Table 3:** Blood and urinary electrolytes concentrations in experimental groups

Group	sNa	sK	sCl	sCr	UV	UNaV	CNa	FENa	UaldV	UKV	CK
Salt solution	145.3 ± 0.9	4.05 ± 0.23	102.0 ± 0.8	0.21 ± 0.01	6.68 ± 5.55	1.77 ± 0.85	12.2 ± 5.8	1.50 ± 0.46	8.42 ± 4.76	9.55 ± 2.72	234 ± 59
Nenrin	144.7 ± 0.8	4.08 ± 0.27	101.7 ± 0.6	0.21 ± 0.01	7.96 ± 3.77	2.27 ± 0.58*	15.7 ± 4.0	1.38 ± 0.28	9.76 ± 3.14	12.5 ± 3.17	312 ± 88*
Shinki	145.0 ± 1.1	4.08 ± 0.21	102.0 ± 1.2	0.21 ± 0.02	7.22 ± 4.48	1.90 ± 0.55	13.1 ± 3.8	1.44 ± 1.02	9.12 ± 3.42	9.12 ± 4.02**	225 ± 101**

Salt solution, group given salt solution; nenrin, Marukome Nenrin group; and shinki, Marukome shinki miso rich in ACE inhibitory activity miso group. Values are expressed as means ± SD (*n* = 10 in each group). Differences were assessed by one-way analysis of variance (ANOVA) followed by Fisher’s least significant difference post-hoc test (LSD test)

*sNa* serum sodium concentrations, *sK* serum potassium concentrations, *sCl* serum chloride concentrations, *sCr* serum creatinine concentrations, *UV* urine volume (mL/100 gBW/day), *UNaV* urinary excretions of sodium (mEq/100 gBW/day), *CNa* sodium clearance (mL/100 gBW/day), *FENa* fractional excretion of sodium (%), *UaldV* urinary excretions of aldosterone (ng/day), *UKV* urinary excretions of potassium (mEq/100 gBW/day), *CK* potassium clearance (mL/100 gBW/day

*P  < 0.05 vs salt solution group, **P  < 0.05 vs nenrin miso group

Shinki miso influenced neither urinary osmolality clearance nor free water clearance, whereas nenrin miso increased osmolality clearance compared to the salt solution (Table 4). In addition, free-water clearance was decreased in the nenrin miso group, suggesting that rats fed nenrin miso excreted more concentrated urine than rats fed the salt solution.

**Table 4:** The effects of Marukome shinki miso on urinary osmolality and free-water clearance

Group	sOsm	Uosm	Cosm	CH_2_O
Salt solution	299.8 ± 2.6	1988.7 ± 789.0	31.9 ± 11.4	−25.2 ± 7.1
Nenrin	298.4 ± 2.0	1766.7 ± 629.8	40.4 ± 9.5*	−32.4 ± 6.8*
Shinki	298.3 ± 2.4	1794.2 ± 886.1	32.2 ± 7.1	−25.0 ± 7.5

Salt solution, group given salt solution; nenrin, Marukome Nenrin miso group; and shinki, Marukome shinki miso rich in ACE inhibitory activity miso group. Values are expressed as means ± SD (*n* = 10 in each group). Differences were assessed by one-way analysis of variance (ANOVA) followed by Fisher’s least significant difference post-hoc test (LSD test)

*sOsm* plasma osmolarity (mOsm/kg H_2_O), *Uosm* urinary osmolarity (mOsm/kg H_2_O), *Cosm* osmolarity clearance (mL/100 gBW/day), *CH*_*2*_*O* free-water clearance(mL/100 gBW/day)

*P < 0.05 vs salt solution group

**Fig. 5:**
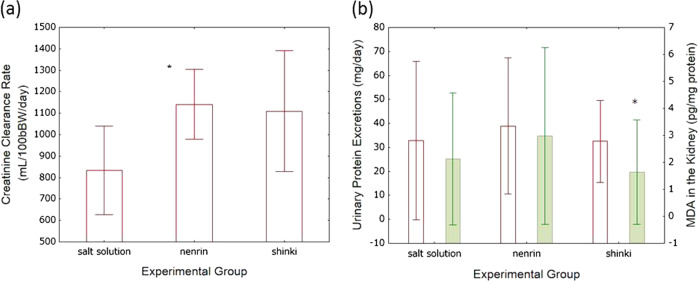
Effects of shinki miso on renal function in SHRSP/Izm. Creatinine clearance rates are shown in graph (**a**). The effects of miso on urinary protein excretion and oxygen stress are shown in graph (**b**). Salt solution, group given salt solution; nenrin, nenrin miso group; shinki, shinki miso rich in ACE inhibitory activity group. The values in the graph represent the means ± SD (*n* = 10 in each group). Differences were assessed with Mann–Whitney *U* tests. *P < 0.05 vs salt solution group

Urinary creatinine clearance was 33% higher in the shinki miso group than in the salt solution group; however, the difference was not statistically significant. Creatinine clearance was 36% higher in rats fed nenrin miso than in rats fed salt solution only (Fig. 5a). There were differences in urinary protein excretion, a target of angiotensin II in the kidney, among the three groups, but the differences were not significant (Fig. 5b).

#### Shinki miso and oxidative stress in the kidney

Since angiotensin II levels may decrease due to the ACE inhibitory activity of miso, which could reduce NADPH oxidase activity and oxidative stress, we tested whether the new miso decreased oxidative stress in the kidneys by measuring MDA production [14–16]. Shinki miso reduced MDA formation compared to the salt solution (Fig. 5b). Moreover, MDA formation in the kidney was positively correlated with urinary protein excretion (*r* = 0.47, *P* < 0.05).

#### Effects of shinki miso on urinary catecholamine excretion

There were no differences in urinary catecholamine excretion between the shinki miso and salt solution groups (Table 5). However, rats in the nenrin miso group excreted higher levels of urinary noradrenaline and dopamine than those in the salt solution group, although the differences were not significant.

**Table 5:** Effects of Marukome shinki miso on catecholamine excretions in urine

Group	UadrV	UnorV	UdopaV
Salt solution	3.75 ± 2.67	45.1 ± 48.6	305.1 ± 259.7
Nenrin	4.32 ± 1.91	58.5 ± 52.3	446.9 ± 255.2
Shinki	4.56 ± 2.49	36.9 ± 15.8	404.1 ± 235.2

Salt solution, group given salt solution; nenrin, Marukome Nenrin miso group and; shinki, Marukome shunki miso group. Values are expressed as means ± SD (*n* = 10 in each group)

*UadrV* urinary excretions of adrenaline (μg/100 gBW/day), *UnorV* urinary excretions of noradrenaline (μg/100 gBW/day), *UdopaV* urinary excretions of dopamine (μg/100 gBW/day)

#### Combined effects of shinki miso and nenrin on salt-induced hypertension in Dahl rats

**Fig. 6:**
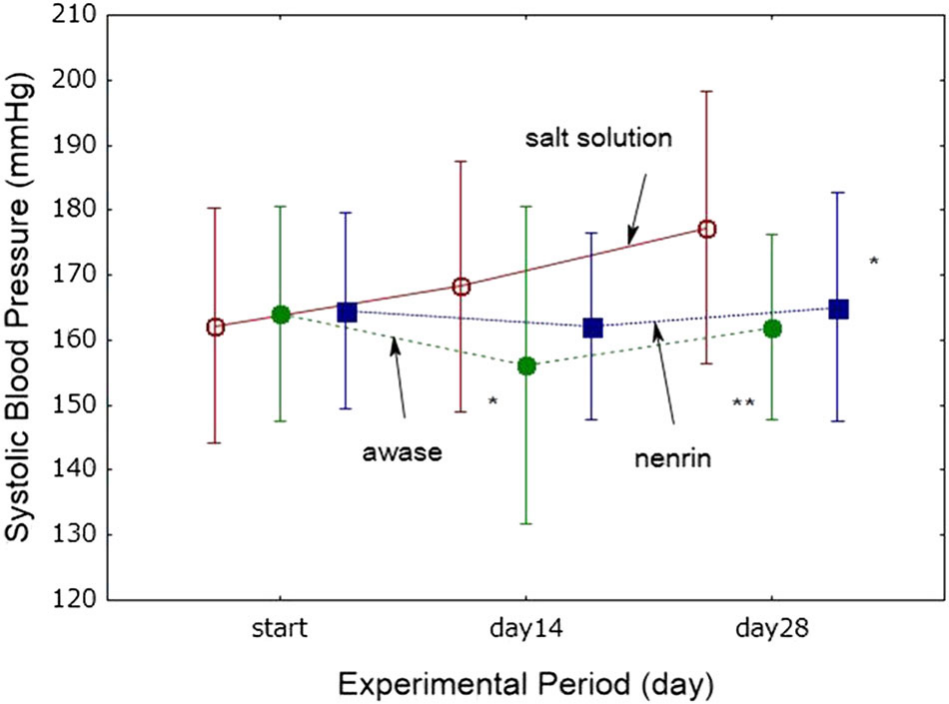
Combined effects of shinki miso and nenrin on salt-induced hypertension in Dahl rats. Salt solution, group given salt solution; awase, group given a combination of shinki and nenrin miso; nenrin, group given nenrin miso. Differences were analyzed by one-way ANOVA followed by Fisher’s LSD test. The values in the graph represent the means ± SD (*n* = 8 in each group). *P < 0.05, **P < 0.001 vs salt solution group

SBP increased in a time-dependent manner in Dahl S rats given the salt solution (Fig. 6). This increase was time-dependently attenuated in rats fed nenrin miso, with a statistically significant difference in the nenrin miso group relative to the salt solution group on day 28. Similarly, in rats that consumed a combination of nenrin and shinki miso, SBP was lower on days 14 and 28 than that in the salt solution group.

**Fig. 7:**
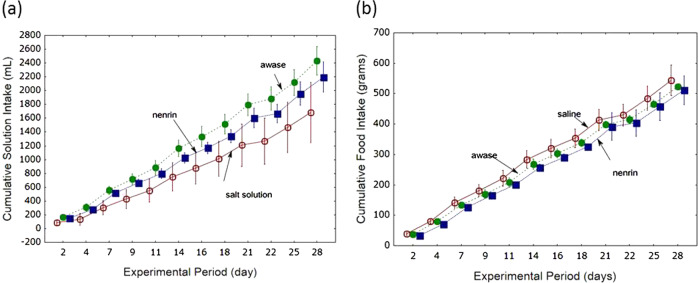
Cumulative solution intake and food consumption in Dahl salt-sensitive rats. The cumulative solution intake during the experiment is shown in graph (**a**), and food consumption is shown in graph (**b**). Salt solution, group given salt solution; awase, group given a combination of shinki and nenrin miso; nenrin, group given nenrin miso. The values in the graph represent as the means ± SD (*n* = 8 in each group). Differences among groups in the cumulative amount of solutions consumed were assessed using repeated measures analysis of variance with Fisher’s LSD test (*P* < 0.0001), and the amount of awase miso consumed was higher than that of nenrin miso (*P* < 0.005). Differences in cumulative food consumption were significant among the three groups (*P* < 0.025); however, there was no difference in food consumption between the awase miso and nenrin miso groups

The cumulative volume of drinking solution consumed increased over time in the three groups (Fig. 7a); however, rats in the miso groups drank more solution than those in the salt solution group, and the amount of solution consumed was higher in the awase group than in the nenrin group. Similarly, food consumption was greater in the miso groups than in the salt solution group (Fig. 7b). However, there was no significant difference in cumulative food consumption between the awase miso and nenrin miso groups.

**Fig. 8:**
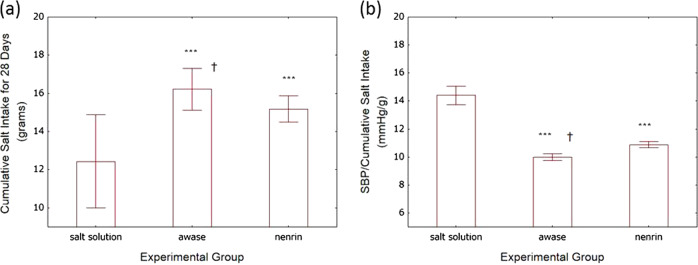
Cumulative salt intake and SBP on day 28 normalized to cumulative salt intake. Based on the amount of solution intake and food consumption over 28 days, the cumulative salt intake was estimated and is shown in graph (**a**). SBP values normalized to cumulative salt intake during the experiment are shown in graph (**b**). Salt solution, group given salt solution; awase, group given a combination of shinki and nenrin miso; nenrin, group given nenrin miso. The values in the graph represent the means ± SD (*n* = 8 in each group). Differences were analyzed by one-way ANOVA followed by Fisher’s LSD test. ***P < 0.001 vs salt solution, †*P* < 0.05 vs nenrin

Based on these data, we estimated the total (cumulative) salt intake during the experiment. The awase miso group consumed more salt (16.2 ± 0.5 g/28 days) than the nenrin (15.1 ± 0.3 g/28 days) and salt solution (12.4 ± 1.2 g/28 days) groups (Fig. 8a). Since salt intake differed among the experimental groups, we normalized SBP on day 28 to cumulative salt intake (Fig. 8b). SBP per gram of salt intake was significantly lower in the nenrin miso group than in the salt solution group. Moreover, SBP per gram of salt intake was significantly lower by 8% in rats fed a combination of shinki and nenrin miso (awase miso) than in rats fed nenrin miso.

**Fig. 9:**
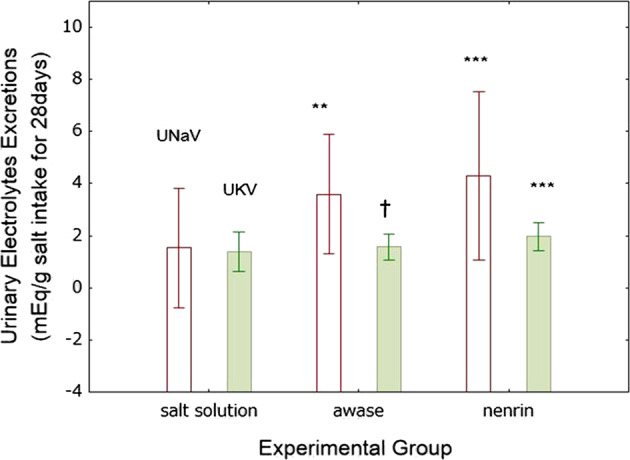
Urinary excretion of sodium and potassium. Salt solution, group given salt solution; awase, group given a combination of shinki and nenrin miso; nenrin, group given nenrin miso. The values in the graph represent the means ± SD (*n* = 8 in each group). Differences were analyzed by one-way ANOVA with Fisher’s LSD test. ***P < 0.001 vs salt solution, †*P* < 0.05 vs nenrin

Urinary Na excretion as a function of salt intake over 28 days was increased in both miso groups relative to the salt solution group; however, there was no difference between the awase and nenrin miso groups (Fig. 9).

## Discussion

The main objective of this study was to produce new miso with hypotensive effects by modifying the fermentation process. Many studies have reported that there is ACE inhibitory activity in enzyme-hydrolyzed fermented soybean or dipeptides derived from soybean [17–19]. To our knowledge, however, there have been few studies in vivo on the involvement of ACE inhibition in BP reduction by miso. The most important finding of the present study is that we have manufactured miso with potent ACE inhibitory activity and that this miso may work as an ACE inhibitor to attenuate hypertension. This finding indicates that the awase miso technique is a strategy to easily produce tasteful miso with antihypertensive effects. In this sense, this is the first study to report the role of ACE inhibition in miso in antihypertensive effects and the benefits of a combination of conventional miso and miso with ACE inhibitory activity.

In this study, we produced miso that has high ACE inhibitory activity (shinki miso) and investigated its hypotensive properties in SHRSP/Izm, a genetic model of spontaneous hypertension [1, 2, 5–7]. These rats are prone to cerebral stroke and show enhanced activation of the RAS and adrenergic nervous system. The most important finding of the present study was that this newly manufactured miso with potent ACE inhibitory activity attenuated spontaneous hypertension in this model. BP decreases were greater in rats fed shinki miso than in rats fed nenrin miso, which is used for daily cooking in Japan. Shinki miso differs from nenrin miso in terms of the Koji starter that is used and the fermentation period. Our results suggest that a slight modification in the manufacturing process, e.g., the species of Koji starter, and potentiation of ACE inhibitory activity contribute to a greater reduction in BP.

We previously demonstrated that long-term intake of traditional miso attenuates salt-induced hypertension through sodium diuresis or vasodilation in Dahl S rats [1, 2]. We also showed that BP reduction in SHRSP/Izm fed nenrin miso was associated with increased urinary sodium excretion. However, in the shinki miso group, there was no increase in urinary sodium excretion, suggesting that the observed reduction in BP was attributable to a mechanism distinct from sodium regulation in the kidneys.

However, to examine the possible natriuretic effects of miso, we assessed urinary Na excretion in the acute phase and Na space in a chronic study. We estimated the ratio of body weights to cumulative salt intake. In Dahl rats at day 28, the ratios were 25.4 ± 2.3 g BW/g salt intake for the salt solution group, 18.0 ± 1.1 g BW/g salt intake for the awase miso group (*P* < 0.05 vs the nenrin miso group and *P* < 0.001 vs the salt solution group, Mann–Whitney *U* test), and 20.1 ± 1.4 g BW/g salt intake for the nenrin miso group (*P* < 0.001 vs the salt solution group, Mann–Whitney *U* test). In the nenrin and awase miso groups, the ratios were significantly lower than that in the salt solution group. This may be explained by a reduction in body fluid space, because the average body weights differed by only 5%. In particular, body weight did not differ between the awase and nenrin miso groups, so the difference in the ratio may be due to a decrease in body fluid space in the awase miso group. However, our findings do not provide direct evidence regarding body fluid space on a long-term basis.

It is conceivable that natriuresis due to nenrin miso and RAS inhibition by shinki miso synergistically decrease salt-induced hypertension. To test this hypothesis, we examined whether the combination of nenrin and shinki miso (awase miso) affected salt-induced hypertension in Dahl S rats and found that BP reduction in the awase miso group was greater than that in the nenrin miso group when SBP was normalized to the amount of salt intake (Fig. 8b). There were no differences in urinary Na excretion between the two miso groups, suggesting that the addition of shinki miso enriched in ACE inhibitory activity enhanced the hypotensive effects of nenrin miso not by promoting natriuresis but through mechanisms related to vasodilation [1, 2].

ACE inhibition and reduced angiotensin II concentrations in blood are presumed to reduce aldosterone secretion, thereby decreasing sodium reabsorption in aldosterone-sensitive distal tubules. However, urinary aldosterone excretion was not diminished by shinki miso, and potassium clearance was unaltered in SHRSP/Izm. The reason for this finding is unclear; however, it is known that suppression of aldosterone by ACE inhibitors can be attenuated even while BP remains controlled [14, 15]. This so-called aldosterone escape may have occurred in the shinki miso group.

In the present study, we did not measure plasma angiotensin concentrations due to the limited amount of blood samples, nor did we determine ACE activity in the SHRSP/Izm study. In the awase miso study, ACE activity was 12.81 ± 4.47 IU/L for the salt solution (*n* = 4), 16.68 ± 6.98 IU/L for nenrin miso (*n* = 3), and 12.80 ± 4.43 IU/L for awase miso (*n* = 5). ACE activity tended to be elevated in nenrin miso compared to the salt solution and may have shifted toward that of the salt solution group when nenrin miso was combined with shinki miso. However, the difference was not significant, and the findings are not conclusive because of the limited samples available to determine ACE activity.

Approximately 50–100 mL of 5% miso solution (2.5–5 g of miso) was consumed by each rat per day. Considering that the average body weight of a rat was 250 g and that the body fluid accounts for 60% of body weight in general, the concentration of miso components in the blood was estimated to be 10–20 mg/g BW or approximately 16–33 mg/mL body fluid. This estimate is much higher than the IC_50_ of shinki miso (0.23 mg/mL), suggesting that shinki miso consumption was enough to suppress the RAS in SHRSP/Izm and Dahl S rats. Data on the bioavailability of active substances were not determined except for salt. We have found that salt in miso is absorbed almost completely through the gastrointestinal tract. However, we could not determine the bioavailability of the ACE inhibitory substance(s), and estimation remains to be performed.

Major antihypertensive effects may be caused by decreased angiotensin II and subsequent vasodilation or facilitation of Na excretion in the kidney. However, it is technically difficult to directly prove the relationship between plasma angiotensin II reduction and these events. Since renal oxygen stress and aortic wall thickness are mediated by angiotensin II upregulation, however, decreased renal oxygen stress and aortic wall thickness probably reflect decreases in plasma angiotensin II, consistent with the findings of a previous study on an ACE inhibitor, alacepril [20]. Moreover, we demonstrated in a Dahl S rat study that salt sensitivity was slightly but significantly decreased through Na diuresis when shinki with ACE inhibitory activity was combined with nenrin miso. Taken together, the results suggest that it is conceivable that the RAS was downregulated by shinki miso under the experimental conditions in the present study.

We demonstrated that oxidative stress in the kidneys of SHRSP/Izm was lower in rats fed shinki miso that had high ACE inhibitory activity than in those that consumed a nenrin miso solution. Angiotensin II generates oxygen radicals through activation of NADPH oxidase [16, 21, 22]. The decreased oxidative stress in the shinki miso group could reflect inhibition of the RAS in the kidneys [23]. We also demonstrated that the aortic wall weight of SHRSP/Izm decreased by 8.7% in the shinki miso group compared to the salt solution group but was unaltered in the regular miso group. This could be explained, in part, by regional RAS inhibition by shinki miso. Moreover, ACE inhibition slows bradykinin degradation and enhances generation of prostacyclin and nitric oxide [23–25], which dilate vessels and reduce vascular smooth muscle cell proliferation [26–28]. These mechanisms could contribute to remodeling of vascular walls in the shinki miso group.

In our previous studies, rats were fed miso that was fermented for 3 or more months [1–3]; urinary sodium excretion and dopamine secretion were higher in these rats than in those that consumed salt solutions. In the present study, we compared the effects of miso fermented for 46 and 5 days. Our results suggest that the fermentation period is critical for sodium handling in the kidneys. However, the precise manner in which the fermentation period provides health benefits remains to be investigated.

### Perspectives

In summary, we showed that a new miso with high ACE inhibitory activity (Marukome shinki miso) has antihypertensive effects in spontaneous and salt-induced hypertension. The antihypertensive mechanisms of shinki miso differed from those of regular commercially available miso (Marukome Nenrin) and potentiated the antihypertensive properties of the regular miso in salt-induced hypertension. New types of miso enriched in specific ingredients with health benefits to humans could have broad applications in the food culture of Japan.
